# REM Sleep EEG Activity and Clinical Correlates in Adults With Autism

**DOI:** 10.3389/fpsyt.2021.659006

**Published:** 2021-06-08

**Authors:** Katia Gagnon, Christianne Bolduc, Laurianne Bastien, Roger Godbout

**Affiliations:** ^1^Sleep Laboratory and Clinic, Hôpital en santé mentale Rivière-des-Prairies, Montréal, QC, Canada; ^2^Departement of Psychiatry, Université de Montréal, Montréal, QC, Canada; ^3^Departement of Psychology, Université de Montréal, Montréal, QC, Canada

**Keywords:** autism, ADI-R, REM sleep, qEEG, EEG distribution

## Abstract

We tested the hypothesis of an atypical scalp distribution of electroencephalography (EEG) activity during Rapid Eye Movement (REM) sleep in young autistic adults. EEG spectral activity and ratios along the anteroposterior axis and across hemispheres were compared in 16 neurotypical (NT) young adults and 17 individuals with autism spectrum disorder (ASD). EEG spectral power was lower in the ASD group over the bilateral central and right parietal (beta activity) as well as bilateral occipital (beta, theta, and total activity) recording sites. The NT group displayed a significant posterior polarity of intra-hemispheric EEG activity while EEG activity was more evenly or anteriorly distributed in ASD participants. No significant inter-hemispheric EEG lateralization was found. Correlations between EEG distribution and ASD symptoms using the Autism Diagnostic Interview-Revised (ADI-R) showed that a higher posterior ratio was associated with a better ADI-R score on communication skills, whereas a higher anterior ratio was related to more restricted interests and repetitive behaviors. EEG activity thus appears to be atypically distributed over the scalp surface in young adults with autism during REM sleep within cerebral hemispheres, and this correlates with some ASD symptoms. These suggests the existence in autism of a common substrate between some of the symptoms of ASD and an atypical organization and/or functioning of the thalamo-cortical loop during REM sleep.

## Introduction

Autism spectrum disorder (ASD) is a neurodevelopmental disorder characterized by impairments of social communication and restrictive/repetitive behaviors (1). An atypical brain developmental curve during critical periods is thought to be involved in the physiopathology of ASD, including brain overgrowth in frontal and temporal areas during the early ages followed by a decline of cortical gray matter thickness during adolescence and young adulthood (2, 3).

Quantitative electroencephalography (QEEG) during wakefulness has been extensively used to characterize brain functioning and its relation to autistic symptoms in ASD children and adolescents (4). However, given the age-specific physiopathology of brain atypicalities in ASD, these results may not apply to adults. There are only two QEEG studies in adults with ASD, and even if comparable methods were used, results are inconsistent (5, 6). An eyes-closed QEEG study comparing ASD adults and control aged between 18 and 38 years found group differences in frontal, pre-frontal, parietal and occipital areas, and less relative alpha activity across all these regions (6). Conversely, using the same eyes-closed recording protocol, Mathewson et al. (5) found no group differences between ASD and typically developing adults on alpha activity while in eyes-open procedure they observe more alpha activity in posterior areas in ASD individuals. These inconsistencies can be explained by the presence of psychiatric comorbidities (6), medications (5, 6), and a wide range of IQ (from 64 to 136) (5).

Resting-state QEEG during wakefulness is also sensitive to experimental settings, including environmental and sensorial inputs (4, 7). Because sensory integration problems are common in autistic individuals with an estimated rate of 90% (8), the experimental environment and sensorial input could influence EEG activity (9). These confounding factors can be controlled by recording the EEG during Rapid Eye Movement (REM) sleep because it is a time of decreased influence of peripheral sensorial input (10, 11) during which a spontaneous and endogenous activation of neural networks occur, making it an ideal condition to study the brain activity.

Sleep studies have shown decreased slow-wave sleep together with atypical non-REM sleep EEG patterns in children and adults on the autism spectrum compared to neurotypical participants, including a decreased density and/or deviant topographical distribution of sleep spindles (12–16), K-complexes (15), and EEG slow-waves (17–21). Studies on REM sleep have not disclosed REM sleep architecture atypicalities in autism but QEEG showed significantly lower spectral power for beta frequency over parietal and occipital recording sites compared to neurotypical controls (22, 23). Specifically in children on the autism spectrum, REM sleep theta spectral power was found to be lower compared to neurotypicals for the left parietal recording sites in one study (23) but not in another (24). In adults with ASD, QEEG analyses disclosed lower NREM sleep delta frequency power over parieto-occipital recording sites but not in the frontal areas as expected (19), suggesting an atypical recruitment of frontal areas. Taken together these results suggest an atypical developmental pattern of the thalamocortical network in children and adults on the autism spectrum.

In order to further test the functioning of the thalamocortical loop in ASD, the present study investigated the topography and distribution of EEG activity along the antero-posterior and transverse axes during REM sleep using a simple but innovative method using spectral power relative distribution. We expected that high-functioning ASD adults would show less beta activity on parietal and occipital electrodes compared to neurotypical adults. Based on previous results on EEG topography in adults with ASD (19, 22), we hypothesized that REM sleep EEG spectral power of high-functioning ASD adults would be anteriorly biased, i.e., higher in anterior than posterior recording sites. Because children with ASD showed an inter-hemispheric left asymmetry in the temporal area (25), we parsimoniously expected the same in adults. To assess the functional consequences of the expected EEG findings, we also explored whether atypical QEEG ratios were statistically associated with scores obtained by the same participants on ASD symptomatology.

## Materials and Methods

### Participants

Seventeen ASD participants and 15 neurotypical (NT) controls of similar chronologic age and full-scale IQ (FSIQ) participated in this study (Table 1). Individuals with ASD were recruited from the specialized autism clinic of a tertiary care hospital. The diagnosis was based on the Autism Diagnostic Interview-Revised (ADI-R) (26), conducted by a psychiatrist and confirmed by an explicit assessment of DSM-IV criteria (American Psychiatric Association, 1994) through direct observation with the Autism Diagnostic Observation Schedule (27) and/or full clinical investigation. Only participants with a full scale intelligence quotient of at least 80, as indicated by their results on the Wechsler Adult Intelligence Scale, 3rd Edition (28), were included. Exclusion criteria were a complaint or a diagnosis of a sleep disorder, a chronic or current illness, a recent history of night work, drug abuse or current use of psychoactive drugs, as documented by a home-made questionnaire, evidence of psychopathology, as recorded by an experienced clinical psychologist or psychiatrist. The NT group was recruited through public advertisements distributed to hospital employees, parents and friends of laboratory staff, as well as in francophone colleges and universities in the Montréal area. All participants received financial compensation for their involvement in this research. The guidelines of the Declaration of Helsinki were followed by obtaining informed consent to participate, and the research project was approved by the Ethical Review Board of Rivière-des-Prairies Hospital.

**Table 1:** Demographic, biometric, and clinical characteristics.

	**NT**	**ASD**	**Student-*t*/*X*^**2**^*p*-values**
Participants (*n*)	16	17	-
Sex (% male)	93.8	94.1	0.93
Age (years)	20.4 ± 4.6	21.6 ± 3.7	0.40
Manual dominance (L/A/R)	1/0/14	3/2/12	-
Cephalometry			
Circumference (cm)	37.5 ± 2.0	37.1 ± 1.6	0.39
Sagittal (cm)	35.5 ± 1.6	36.1 ± 2.2	0.38
Coronal (cm)	56.4 ± 1.9	57.6 ± 2.4	0.12
Intellectual quotient			
Full IQ	111.2 ± 11.6	103.6 ± 12.05	0.09
Performance IQ	109.2 ± 12.1	102.7 ± 14.0	0.19
Verbal IQ	111.0 ± 10.1	103.2 ± 16.2	0.14
ADI-R			
Social	-	20.4 ± 3.7	-
Communication	-	14.9 ± 4.6	-
Interest	-	7.1 ± 3.4	-

Results are presented as mean ± standard deviation. IQ, intelligence quotient; L, left handed; A, Ambidextrous; R, right handed. ^*^p < 0.05; - = statistical test not performed.

### Polysomnography Recording

Participants were asked to keep a regular sleep-wake schedule for 14 days before coming to the laboratory, and to refrain from napping during the day before the recording; none were regular nappers. Beverages containing caffeine and alcohol were not allowed after 12:00 noon.

Participants were recorded for two consecutive nights. Recordings were performed in sound attenuated, well shaded, temperature controlled bedrooms at the sleep laboratory of the Hôpital en santé mentale Rivière-des-Prairies. Participants had the possibility to go to bed and rise at their preferred time. All data reported in this paper come from the second night in the laboratory. The first night was used for adaptation to the recording equipment, the procedures, and the environment. During the first night, respiratory flow and effort were monitored using oronasal cannula and thoraco-abdominal strain gauges, respectively, and transcutaneous finger pulse oximetry was used to monitor arterial blood oxygen saturation. Bilateral anteror tibilais EMG was recorded to detect periodic movements in sleep. EEG was recorded with a 22-electrodes montage positioned according to the international 10–20 system with linked earlobes references (29, 30). A Grass Neurodata Model 15 Acquisition System was used for recording, and signals were digitized at a sampling rate of 256 Hz using Harmonie 5.0B software (Stellate, Montréal, Canada). Filter settings and amplification factors were 1/2 amplitude low frequency filter = 0.3 Hz, 1/2 amplitude high frequency filter = 100 Hz, gain x 1,000 = 20.

Sleep was scored according to standard methods, using 20-s epochs (31). Sleep latency was defined as the occurrence of the first 10 consecutive min of stage 1 sleep or the first epoch of any other sleep stage after lights off. REM sleep periods occur when rapid eye movements, muscular atony, and low amplitudes mixed frequencies were present for more than 50% of an epoch. Ten minutes of non-REM sleep was required between two different REM sleep periods. REM sleep latency was defined as the interval between sleep latency and the first REM sleep epoch. Sleep efficiency was computed as the percentage of sleep time between sleep latency and the final awakening. Polysomnographic recordings did not reveal any patients with an Apnea-Hypopnea Index of 10 or higher nor a Periodic Leg Movement Index of 10 or higher. No cases with epileptiform EEG, with or without corresponding behavioral manifestations, were encountered.

### REM Sleep EEG Spectral Activity Data Acquisition

REM sleep EEG samples of 15 four-s segments were taken in similar proportions through the first four REM sleep periods, totaling 60 s of artifact-free EEG signal for each of the 16 electrodes used, i.e.,: Fp_1_, Fp_2_, F_3_, F_4_, F_7_, F_8_, C_3_, C_4_, T_7_, T_8_, P_3_, P_4_, P_7_, P_8_, O_1_, and O_2_. EEG samples were taken during ocular (EOG) quiescent periods. Particular attention was paid to discard EEG segments containing EMG artifacts. EEG samples were Fast-Fourier transformed with a resolution of 0.25 Hz and cosine window smoothing. Spectral analysis was performed on the total frequency and four frequency bands were extracted using a commercially available package (Harmonie 5.0B, Stellate, Montréal): Delta (0.75–3.75 Hz), Theta (4.0–7.75 Hz), Alpha (8.0–12.75 Hz), and Beta (13.0–19.75 Hz).

### REM Sleep EEG Distribution Calculation of Intrahemispheric Ratios

Intrahemispheric antero-posterior EEG activity ratios between proximal recording sites (i.e., immediately neighboring electrodes) and between distal recording sites were computed with the following formula: [(posterior electrode – anterior electrode)/(posterior electrode + anterior electrode) ^*^ 100]. Positive values thus indicate a posterior bias, i.e., that spectral power is higher over the posterior recording site compared to the anterior recording site, relative to the total power expressed by the sum of the two; negative values consequently indicate an anterior bias distribution. This method was previously used by us in neurotypical adults on wake and REM sleep EEG (32).

### REM Sleep EEG Distribution Calculation of Interhemispheric Ratios

Interhemispheric activity ratios between left and right homologous electrodes were computed with the following formula: [(right electrode – left electrode)/(right electrode + left electrode) ^*^ 100]. Positive values thus indicate a right bias and negative values indicate a left bias distribution.

### Statistical Analysis

#### Demographic and Sleep Characteristics

Statistical analyses were performed with SPSS 25.0 (SPSS Science, Chicago, Illinois, USA). Statistical significance was set at *p* < 0.05. Student *t*-tests and Person Chi-squares were used to test group differences (ASD vs. NT) on demographics (age, sex, IQ) and sleep variables.

#### REM Sleep EEG Spectral Activity

One-way ANOVAs were used to test differences between ASD and NT groups on log-transformed absolute power values for each electrode for the four frequency bands (Delta, Theta, Alpha, and Beta), and total activity. Levene's test for homogeneity of variance were systematically applied. Effect sizes were calculated using Partial Eta Squares ($\eta_{p}^{2}$) and full data are provided in the Supplementary Material. To compensate for multiple tests performed, only results with large effect sizes were considered as statistically significant ($\eta_{p}^{2}\;\geq$ 0.14) (33).

#### REM Sleep EEG Intrahemispheric and Interhemispheric Ratios

Group comparisons (ASD vs. NT) on each frequency band for each electrode pairs were done using one-way ANOVAs. Levene's test for homogeneity of variance were also applied. Effect sized were determined with Partial Eta Squares As indicated by Lakens (33), only results with large effect size, i.e., $\eta_{p}^{2}\;\geq \;$0.14, are reported as statistically significant (33).

#### The Relationship Between REM Sleep Atypical EEG Activity Distribution and ASD Symptoms

The association between atypically-distributed QEEG ratio values and ADI-R subscales scores (social, communication, restricted interests) was assessed with Pearson correlation coefficients.

## Results

No significant between-group differences were found in demographic data (Table 1). We found significantly longer sleep latency and a trend for less REM sleep periods in the ASD group compared to the NT group (Table 2).

**Table 2:** Sleep characteristics in 17 young autistic adults (ASD) and 16 neurotypical controls (NT).

	**NT**	**ASD**	**Student-*t p*-values**
Sleep Latency (min)	8.6, 5.1	18.0, 14.7	0.02^*^
TST (min)	459.4, 49.5	436.1, 89.9	0.38
Awakenings (number)	22.6, 17.2	25.1, 15.9	0.68
Awakenings (min)	24.7, 29.8	23.5, 19.2	0.89
Efficiency (%)	95.0, 5.8	95.0, 4.2	0.99
Stage 1 (%)	5.0, 3.0	6.1, 3.6	0.34
Stage 2 (%)	59.0, 7.9	61.1, 8.5	0.50
Stage 3 (%)	8.3, 2.9	6.1, 3.5	0.07
Stage 4 (%)	5.1, 6.7	2.4, 4.6	0.20
Stage 3+4 (%)	13.4, 7.6	8.6, 6.7	0.07
REM (%)	22.6, 2.8	24.2, 6.8	0.41
REM latency (min)	70.8, 17.0	70.0, 14.8	0.89
REM periods (number)	5.1, 0.6	4.5, 1.0	0.05
REM duration	117.4, 22.0	121.3, 48.2	0.78

*Results are presented as (mean ± standard deviation)*.

*REM, Rapid eye movement; TST, total sleep time; min, minutes*.

* *p < 0.05*.

### Topography of REM Sleep EEG Activity

**Figure 1:**
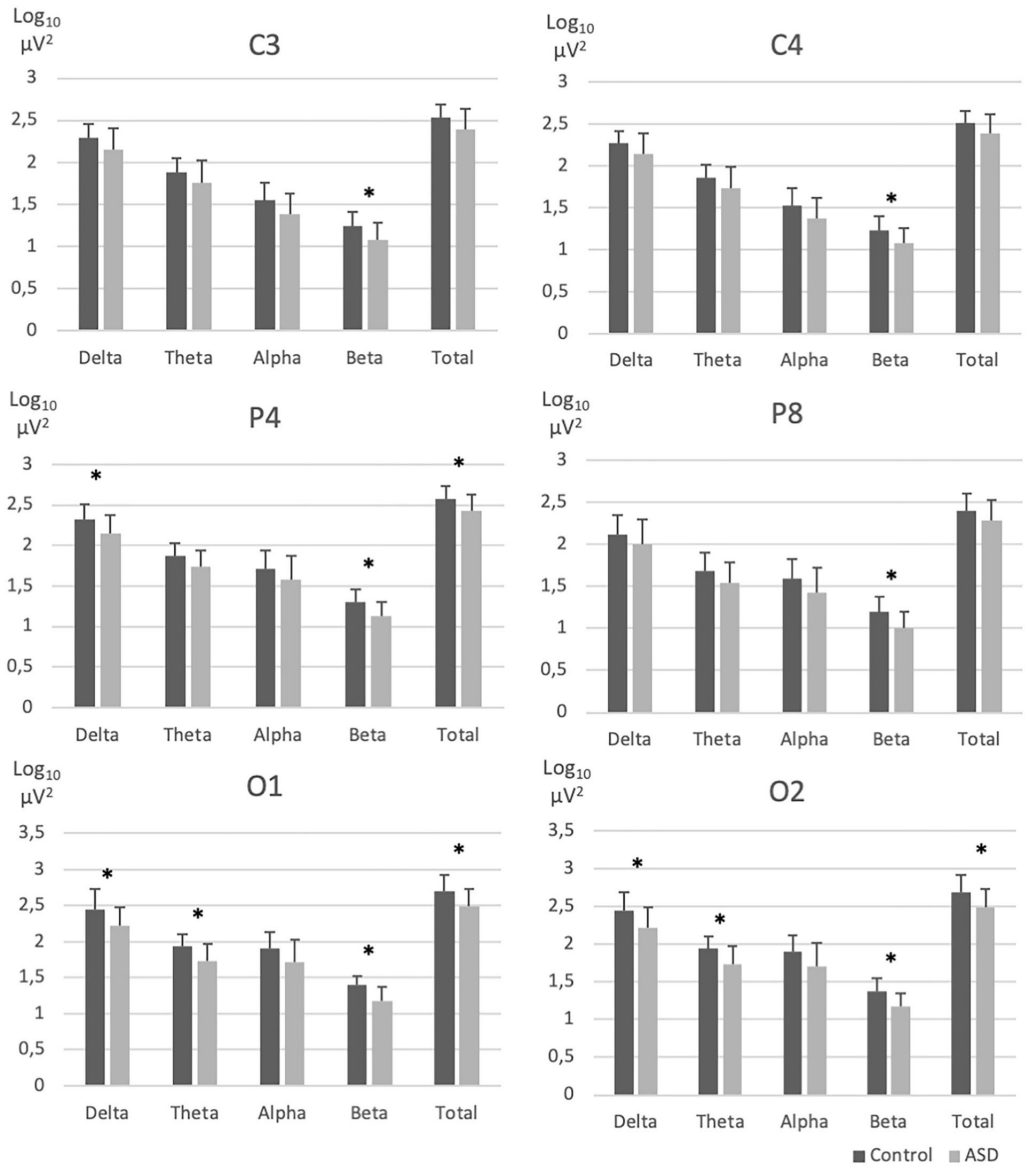
EEG absolute spectral power across frequency bands in electrodes with significant group differences. ASD, Autism spectrum disorder; EEG, Electroencephalography; NT, Neurotypical. Error bars represent standard errors of the means. ^*^p < 0.05.

REM sleep EEG beta and theta activity was significantly lower for the centro-posterior electrodes in the ASD group compared to the NT group (Figure 1 and Supplementary Table 1). More specifically, groups differences appeared on bilateral central electrode [C3 beta (*F*_(1, 30)_ = 6.40, *p* = 0.02); C4 beta (*F*_(1, 30)_ = 6.47, *p* = 0.02)], right parietal area [P4 delta (*F*_(1, 30)_ = 5.24; *p* = 0.03); beta (*F*_(1, 30)_ = 7.79; *p* = 0.009); total (*F*_(1, 30)_ = 5.50; *p* = 0.03), P8 beta (*F*_(1, 30)_ = 7.84, *p* = 0.01)] and bilateral occipital electrodes [O1 beta (*F*_(1, 28)_ = 12.64, *p* = 0.001), theta (*F*_(1, 28)_ = 7.25, *p* = 0.01), delta (*F*_(1, 28)_ = 5.20, *p* = 0.03), total (*F*_(1, 28)_ = 5.79, *p* = 0.02); O2 beta (*F*_(1, 30)_ = 9.60, *p* = 0.004), theta (*F*_(1, 30)_ = 7.25, *p* = 0.01) delta (*F*_(1, 30)_ = 6.10, *p* = 0.02), and total (*F*_(1, 30)_ = 6.16, *p* = 0.02)].

### REM Sleep Distribution of Intrahemispheric Proximal Ratios

**Figure 2:**
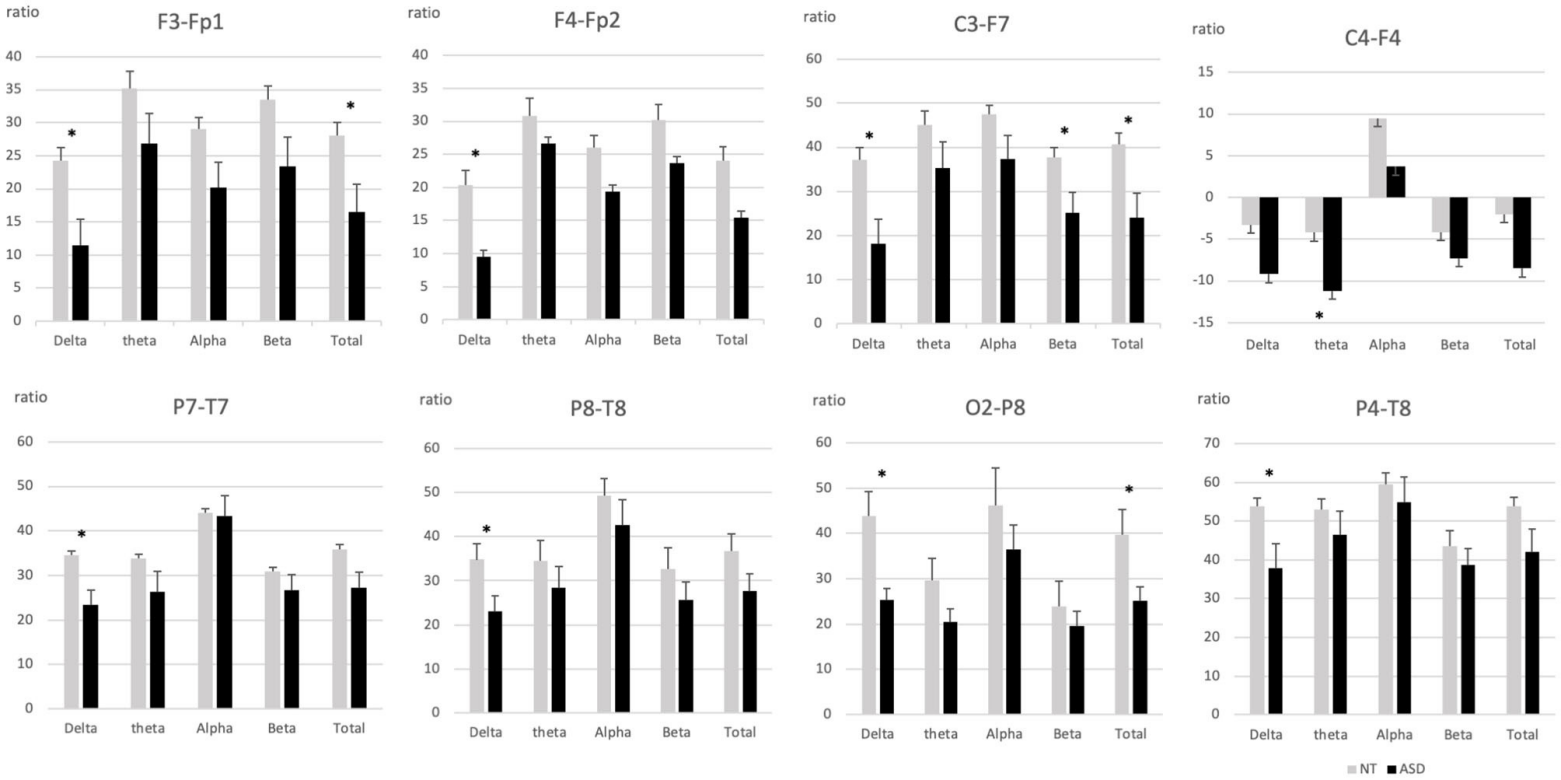
Intrahemispheric proximal ratios across frequency bands in pairs if electrodes with significant group differences. ASD, Autism spectrum disorder; NT, Neurotypical. Error bars represent standard errors of the mean. ^*^p < 0.05.

The NT group generally showed higher ratios on posterior EEG distribution compared to ASD and the ASD group displayed smaller antero-posterior electrodes differences compared to the NT group. Figure 2 highlight the significant positive (posteriorly biased) proximal ratio differences (see Supplementary Table 2 for all results). Significantly different positive proximal ratios were found both frontal/pre-frontal area: F3-Fp1 [delta (*F*_(1, 30)_ = 7.86, *p* = 0.009), total (F_(1, 30)_ = 5.94, *p* = 0.02)] and F4-Fp2 [delta (*F*_(1, 30)_ = 6.65; *p* = 0.015)]. Left centro-frontal area showed posteriorly biased ratios on C3-F7 [delta (*F*_(1, 29)_ = 8.21, *p* = 0.008); beta (*F*_(1, 29)_ = 4.98, *p* = 0.03); total (*F*_(1, 29)_ = 6.43, *p* = 0.017)]. Parieto-temporal electrodes ratios were significantly positive for P4-T8 [delta (*F*_(1, 30)_ = 5.35, *p* = 0.028)], P7-T7 [delta (*F*_(1, 29)_ = 4.52, *p* = 0.042)], and P8-T8 [delta (*F*_(1, 30)_ = 5.48, *p* = 0.026)]. Right occipito-parietal ratio was significant for O2-P8 [delta (*F*_(1, 30)_ = 10.14, *p* = 0.003); total (*F*_(1, 30)_ = 5.81, *p* = 0.022)].

The only case where the ASD group showed a greater ratio than the NT group involved electrodes pairs for which the ASD group displayed a significant anterior polarity, i.e., we found significant negative ratios for the right centro-frontal electrodes C4-F4 [theta (*F*_(1, 30)_ = 4.77, *p* = 0.037)].

### Intrahemispheric Ratios of EEG Activity Between Pairs of Distal Recording Sites

**Figure 3:**
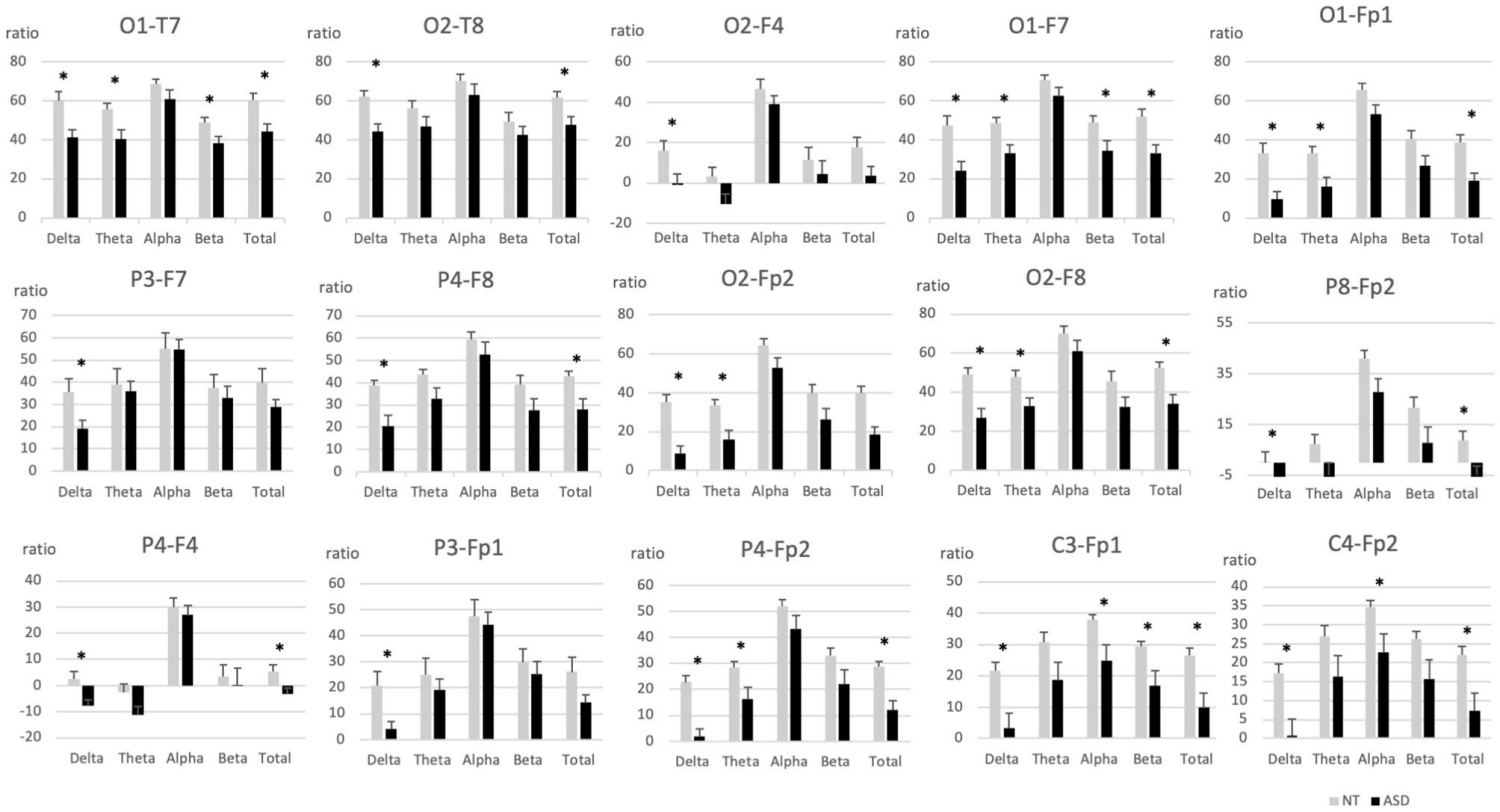
Intrahemispheric distal ratios across frequency bands in pairs of electrodes with significant group differences. ASD, Autism spectrum disorder; EEG, Electroencephalography; NT, Neurotypical. Error bars represent standard errors of the mean. ^*^p < 0.05.

Results showed a higher posterior biased distribution of spectral activity in the group of NT participants compared to the ASD group. Differences between the distal posterior and the anterior electrodes were also lower in the ASD group when ratios had a posteriorly-biased distribution (Figure 3 and Supplementary Table 3).

Group comparisons on intrahemispheric distal ratios showed significant posteriorly-bias differences of the NT group on occipito-temporal electrode pairs O1-T7 [delta (*F*_(1, 28)_ = 10.38, *p* = 0.003); theta (*F*_(1, 30)_ = 6.70, *p* = 0.015); beta (*F*_(1, 30)_ = 5.19, *p* = 0.03); total (*F*_(1, 28)_ = 9.45, *p* = 0.005)], O2-T8 [delta (*F*_(1, 30)_ = 12.98, *p* = 0.001); total (*F*_(1, 30)_ = 6.87, *p* = 0.014)], occipito-frontal O1-F7 [delta (*F*_(1, 28)_ = 10.52, *p* = 0.003); theta (*F*_(1, 28)_ = 7.89, *p* = 0.009); beta (*F*_(1, 28)_ = 5.21, *p* = 0.03); total (*F*_(1, 28)_ = 9.57, *p* = 0.004)], O2-F8 [delta (*F*_(1, 30)_ = 13.29, *p* = 0.001); theta (*F*_(1, 30)_ = 6.93, *p* = 0.013); total (*F*_(1, 30)_ = 9.82, *p* = 0.004)], O2-F4 [total (*F*_(1, 30)_ = 4.76, *p* = 0.037)] and occipito-pre-frontal electrode pairs O1-Fp1 [delta (*F*_(1, 28)_ = 14.05, *p* = 0.001); theta (*F*_(1, 28)_ = 8.56, *p* = 0.007); total (*F*_(1, 28)_ = 11.87, *p* = 0.002)], O2-Fp2 [delta (*F*_(1, 30)_ = 22.78, *p* = 0.000); theta (*F*_(1, 30)_ = 9.35, *p* = 0.005); total (*F*_(1, 30)_ = 17.34, *p* = 0.000)]. Parieto-frontal electrode pairs P4-F8 [delta (*F*_(1, 30)_ = 11.11, *p* = 0.002); total (*F*_(1, 30)_ = 7.05, *p* = 0.013)], P3-F7 [delta (*F*_(1, 29)_ = 5.79, *p* = 0.023)], and parieto-pre-frontal electrode pairs P4-Fp2 [delta (*F*_(1, 30)_ = 29.18, *p* = 0.000); theta (*F*_(1, 30)_ = 5.84, *p* = 0.022); total (*F*_(1, 30)_ = 15.77, *p* = 0.000)], P3-Fp1 [delta (*F*_(1, 30)_ = 7.10, *p* = 0.012)] were also significantly posteriorly biased, as well as centro-pre-frontal electrode pairs C3-Fp1 [delta (*F*_(1, 30)_ = 10.6, *p* = 0.003); alpha (*F*_(1, 30)_ = 5.26, *p* = 0.029); beta (*F*_(1, 30)_ = 5.32, *p* = 0.028); total (*F*_(1, 30)_ = 8.81, *p* = 0.006)], C4-Fp2 [delta (*F*_(1, 30)_ = 9.86, *p* = 0.004); alpha (*F*_(1, 30)_ = 4.84 *p* = 0.036); total (*F*_(1, 30)_ = 7.72, *p* = 0.009)].

Anterior ratios biased results were found on right occipito-frontal electrode pairs O2-F4 [delta (*F*_(1, 30)_ = 5.64, *p* = 0.024)], parieto-frontal P4-F4 [delta (*F*_(1, 30)_ = 8.90, *p* = 0.006); total (*F*_(1, 30)_ = 5.52, *p* = 0.026)] and the right parieto-pre-frontal electrode pair P8-Fp2 [delta (*F*_(1, 30)_ = 6.44, *p* = 0.017); total (*F*_(1, 30)_ = 6.20, *p* = 0.019)].

### Interhemispheric Ratios of EEG Activity Between Homologous Pairs of Recording Sites

No significant group differences were found (Supplementary Table 4).

### The Relationship Between ADI-R Scores and Atypical QEEG Intrahemispheric Activity Ratios

**Figure 4:**
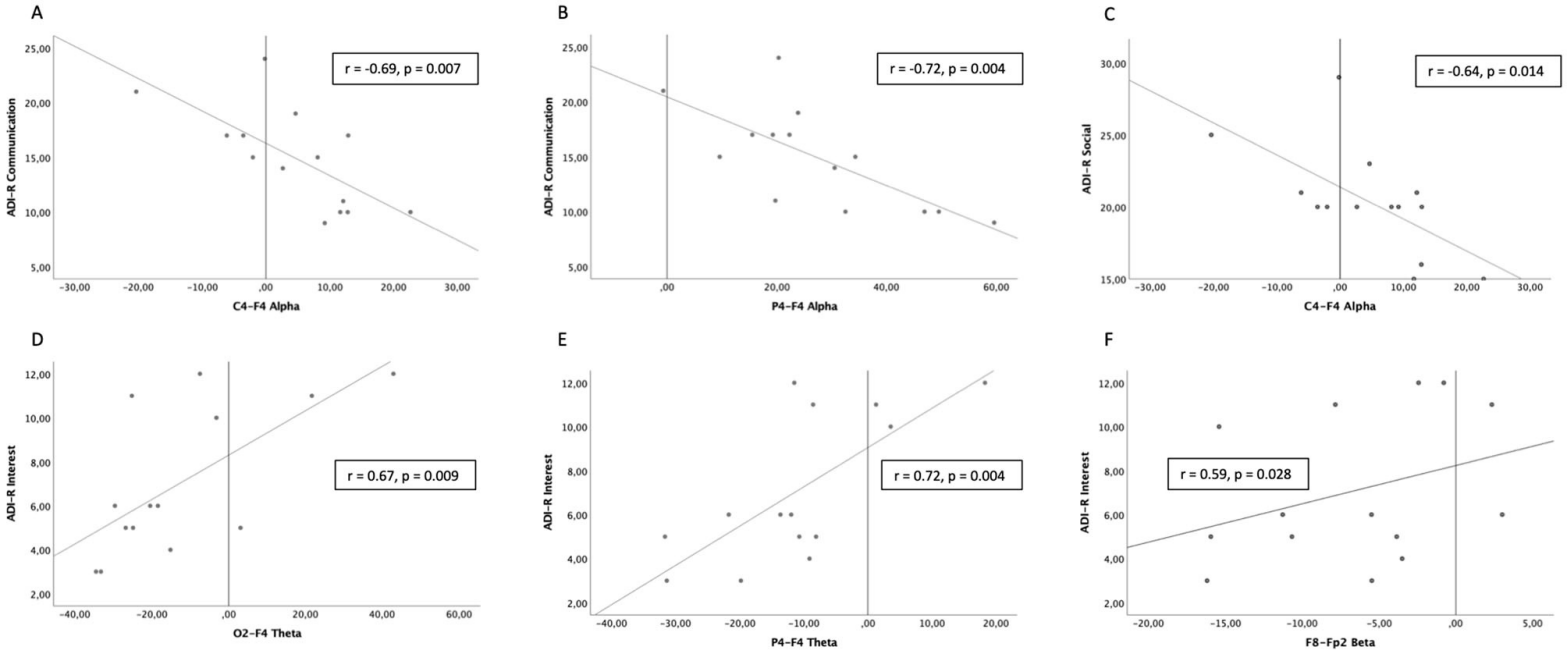
Significant correlations between scores on the Autism Diagnostic Interview-Revised (ADI-R) and intrahemispheric ratios of EEG activity in Autism Spectrum Disorder (ASD) participants. **(A,B)** Communication scores with C4-F4 and P4-F4 activity ratios in the alpha frequency range. **(C)** Social interaction scores with C4-F4 activity ratios in the alpha frequency range. **(D,E)** Restricted interests and repetitive behaviors scores with O2-F4 and P4-F4 activity ratios in the theta frequency range. **(F)** Restricted interests and repetitive behaviors scores with P8-Fp2 activity ratios in the beta frequency range.

According to the previous results, correlations were performed only on the four pairs of electrodes with a significant atypical distribution, namely C4-F4, O2-F4, P4-F4, and P8-Fp2. ADI-R communication scale scores were significantly negatively correlated with the activity ratio between C4-F4 and between P4-F4 recording sites in the alpha frequency range (Figures 4A,B). ADI-R social interaction scores were significantly negatively correlated with the activity ratio between C4-F4 recording sites in the alpha frequency range (Figure 4C). ADI-R interest scale scores were positively correlated with the activity ratio between O2-F4 and between P4-F4 recording sites in the theta frequency range (Figures 4D,E) and also positively between P8-Fp2 recording sites in the beta frequency range (Figure 4F).

## Discussion

This study compared REM sleep EEG activity topography and distribution between ASD adults and a NT adult comparison group. The hypothesis of a lower EEG activity in the ASD group over occipital and parietal recoding sites compared to the NT group was supported, with the additional finding of a lower EEG activity over central recording sites. As expected, the intra-hemispheric EEG activity was found to be posteriorly biased in the NT group while it was more evenly or anteriorly distributed in ASD participants. We did not find, however, any interhemispheric group differences. Finally, the higher anteriorly-biased ratios over the right centro-frontal and parieto-frontal areas were associated with higher scores on the communication scale of the ADI-R, whereas the higher anteriorly-biased ratios of the right parieto-frontal and occipito-frontal areas were associated with lower scores on the restricted interests scale.

### EEG Topography

Cerebral lateralization in ASD has been a topic of discussion since the mid 1970s (34). The first peer-reviewed journal article on EEG lateralization in autism is possibly that of Dawson et al. (35), with evoked potentials recorded during wake with three electrodes Cz (vertex), midway between C3 and T5 (left hemisphere), and mid-way between C4 and T6 (right hemisphere) and using a simple, imprecise formula (right minus left latencies and amplitudes of evoked responses to a verbal stimulus). The present findings confirm and extend previous results by Daoust et al. (22) in adults and adolescents, because both studies found decreased in REM sleep EEG total power in occipital recording sites of the ASD group. In the present study, additional differences were found over central recording sites. Because the study of Daoust et al. (22) included adolescents, the present findings on central recording sites could be related to developmental aspects. As a matter of fact, a recent retrospective cross-sectional structural MRI study showed an increasing intra-regional cortical thickness variability with age in autism, specifically in occipital, parietal and central areas (36). Longitudinal studies in carefully diagnosed persons with ASD and combining EEG with brain imaging are needed to better understand the effect of atypical cortical development patterns on EEG activity in ASD.

One functional meaning of the present results relates to mental activity during REM sleep, i.e., dreaming. Based on questionnaires, we have previously shown that recall of dream content for the past month was lower in ASD adults compared to controls (37),while others have reported that dream recall was associated with increased beta EEG activity over occipital recording sites (38). This is consonant with the fact that, in the present study, beta EEG activity over occipital recording sites was found to be lower in the ASD group. This is also in agreement with the fact that, on one hand, dream narratives collected following REM sleep awakenings in the laboratory were found to be shorter and that the content was impoverished in ASD adults, including fewer emotional elements while (37), on the other hand, emotions in dreams were reported to be positively correlated with a rightly-biased occipital EEG asymmetry (39).

### Relationships Between REM Sleep and Waking EEG Activity

Studies investigated resting-state waking EEG in adults with ASD using an eyes-closed resting-state protocol, which could mimic the sleeping state, found mixed results. Mathewson et al. (5) focused on absolute alpha power frequency and found no group difference over the whole scalp. Murias et al. (6) found frequency-specific group contrasts on relative EEG activity, where anterior and posterior sites generated less alpha activity, which was compensated with relatively more beta and theta activity, respectively. Because EEG oscillations in the theta range is thought to reflect local cortical processing whereas alpha activity would be rather associated with global cortical processing, their results support the existence of a local overconnectivity and large scale under-connectivity in ASD (40).

Conversely to eyes-closed resting-state, REM sleep represent the spontaneous activity of the brain without interference from external stimuli (10). Sleep studies suggested that different frequency bands during sleep could be associated with specific functional aspects of REM sleep; beta activity would reflect the activation of the neuronal network involved in the control of REM sleep (41) whereas theta activity during REM sleep would be associated with cognitive processing (42). Hence, lower beta and theta activity found in centro-parietal-occipital areas in the present study could represent, on one hand, the expression of an atypical network controlling REM sleep and, on the other hand, an impaired REM sleep-related cognitive processing in ASD individuals.

### Lower EEG Power Differences Across the Scalp During REM Sleep in ASD

The distribution of REM sleep EEG activity was mostly posteriorly distributed in NT as well as in the ASD group. Interestingly, ASD individuals had less EEG activity differences between intrahemispheric anteroposterior recording sites compared to NT. This suggests atypicalities in thalamo-cortical and cortico-cortical functioning in ASD (10), which is supported by the recent findings of particularities in cortico-cortical and thalamo-cortical projections. Studies using structural magnetic resonance imaging (MRI) investigated cortical thickness found gray matter reductions in all cortical regions of adults with ASD (43–46). Moreover, also using structural MRI, Ecker et al. (47) showed that cortico-cortical connectivity was locally and globally reduced in ASD. These results suggest that lower cortical EEG activity and its atypical distribution between proximal recording sites in ASD could be explained by gray matter and cortico-cortical connectivity atypicalities.

Longitudinal studies using structural MRI in ASD showed an atypical white matter development from childhood to adulthood, including a significant decrease in the mean volume of white matter (46) as well as an absence of increasing fractional anisotropy with age (48). The latter study also reported that axial diffusivity was decreasing with age in ASD, but not in typically developing individuals. These suggest the presence in ASD of fewer/thinner axons, decreased myelination, and less coherence in the directions of myelination over development, which could influence EEG difference between distal electrodes (48–51).

Taken together, imaging studies support the idea of atypical development of neural circuits through adulthood that could influence REM sleep EEG production and distribution in adult ASD. Atypical gray and white matter development could affect the cortico-cortical and thalamo-cortical neurophysiological activity and lead to an atypical topography of EEG activity as shown here as well as in previous studies on REM sleep (22) and on non-REM sleep EEG (19).

### Anteriorly-Biased EEG Power Distribution in ASD

Higher anteriorly-biased EEG distribution in ASD was found in right parieto-frontal/pre-frontal, occipito-pre-frontal, and centro-frontal areas. While this anteriorly-biased EEG distribution could be explained, at least partly, by a lower EEG spectral power in posterior electrodes the literature rather points toward atypical spectral power in anterior areas. On one hand, studies investigating resting-state networks using MRI, EEG, and magnetoencephalography have shown network atypicalities that included frontal areas, such as the default mode and the sensorimotor networks (52–54). Moreover, EEG overconnectivity was found in bilateral frontal and left parietal areas (53). On the other hand, EEG coherence activity during REM sleep in adults with autism (55) showed a different pattern, with a lower connectivity in the right frontal area while occipital areas were overconnected to other intrahemispheric regions compared to neurotypical controls. This posterior overconnectivity could reflect a greater influence of the white matter between occipital areas and other regions (56), whereas the lower spectral power in occipital areas could be a result of lower gray matter density (57).

### Functional Imbalance of QEEG Distribution in Bilateral Parieto-Occipital and Right Frontal Areas During REM Sleep

Although we found significant differences in slow (Delta, Theta) and high (Beta) EEG frequency distribution, it may be early to interpret the functional specificity of each frequency band at this point but the results can be discussed at the level of functional intrahemispheric QEEG imbalance involving the bilateral parieto-occipital and the right frontal areas. REM sleep plays a key role in memory consolidation, emotional processing (58), and is the physiological support of dreaming (38) (and see EEG Topography, above). During REM sleep, the frontal area is active and has been associated with recent emotional processing and memory (59). Consequently, right frontal EEG activity could represent a greater load of emotional regulation/consolidation during REM sleep in ASD, or an atypical process of emotion regulation/consolidation. Both REM and NREM sleep are implicated in memory consolidation (60, 61) and frontal NREM sleep slow EEG activity is positively associated with the learning index of a sensory-motor procedural task in ASD (19). This support the activation of the frontal area in memory consolidation during sleep in individuals with ASD.

Parieto-occipital areas are supporting the production of visual oneiric content (62, 63). Oneiric content has been found to be less elaborate in ASD after controlling for confounding factors (37). Therefore, lower QEEG in the parieto-occipital areas could reflect the poor quality of visual oneiric content during REM sleep.

### Relationship Between ASD Symptoms and REM Sleep Anteriorly-Biased QEEG Activity Distribution

Anteriorly-biased activity ratios on proximal as well as distal pairs of recording sites were associated with higher scores on the ADI-R social and communication scales (i.e., low social and communication skills) while anteriorly-biased activity ratios on distal pairs of recording sites were associated with lower scores on the ADI-R interest scale (i.e., less restricted interests and repetitive behaviors) in the ASD group. All significant correlations involved EEG activity recorded over the right hemisphere. Interestingly, abilities that are crucial for adapted social interactions (i.e., attribute thoughts and feelings to self and others) and communication (i.e., pragmatic language) are reported to involve the right hemisphere (64–66). Moreover, the right frontostriatal loop has been identified as a potential neuronal substrate of restricted interests and repetitive behaviors in autism (67, 68). To our knowledge, no study has investigated the associations between the ADI-R scales and EEG distribution activity in ASD and the present results adds to the existing literature suggesting that an atypical right hemisphere functioning could contribute to ASD behavior as measured by the ADI-R.

### Strength and Limitations

This research innovates by investigating cortical activity distribution across brain regions using a simple method. Considering sensorial challenge encountered by ASD individuals during the wake state, another strength of the present method is the use of REM sleep EEG to minimize interference from external inputs.

Recent imaging studies have shown increased intraregional cortical thickness variability in individuals with ASD (36, 54). In this context, the relatively small sample studied here could be seen as a limitation. However, the ASD participants were clinically characterized in the most stringent manner, including the absence of medication, intellectual disability, psychiatric or sleep disorder.

## Conclusion

We found an atypical scalp distribution of EEG activity in ASD participants during REM sleep, with less activity in centro-posterior areas and lower EEG power differences across recording sites. Significant higher anterior EEG distribution was shown in ASD and this anterior bias was associated with ASD core symptoms, such as poor communication skills and less restricted interests. Our results provide new and independent evidence of the presence of a relation between EEG activity and the ASD phenotype. Concurrently, this highlights the importance of studying REM sleep EEG activity in subsequent studies that would like to establish a detailed portrait of clinicopathological associations with EEG activity in the ASD population.

## Data Availability Statement

The datasets presented in this article are not readily available because these data are confidential and need an authorization from the ethical review board of the Hôpital Rivière-des-Prairie to be shared. Requests to access the datasets should be directed to comite.ethique.recherche.cnmtl@ssss.gouv.qc.ca.

## Ethics Statement

The studies involving human participants were reviewed and approved by Ethical review board of Hôpital en santé mentale Rivière-des-Prairies. The patients/participants provided their written informed consent to participate in this study.

## Author Contributions

KG contributed to the study design, data analysis and interpretation, manuscript redaction, and revisions. CB contributed to the study conception and design, data collection, data analysis, manuscript redaction, and revisions. LB contributed to study design, data interpretation, manuscript redaction, and revisions. RG contributed to study conception and design, data collection, data analysis, data interpretation, manuscript redaction, and revisions. All authors approved the final version for publication and are accountable for all aspect of the work.

## Conflict of Interest

The authors declare that the research was conducted in the absence of any commercial or financial relationships that could be construed as a potential conflict of interest.
