# Supplementary material for: REM Sleep EEG Activity and Clinical Correlates in Adults With Autism
Source: Front Psychiatry. 2021 Jun 8;12:659006. doi: 10.3389/fpsyt.2021.659006 (PMC8217632; doi:10.3389/fpsyt.2021.659006)
Supplement: Supplementary file 1 [file Data_Sheet_1.docx]

# Supplementary Material

**Table S1.** Group differences in EEG spectral activity.

| **Electrode** | **Frequency band** | **NT** | **ASD** | **F-value** | **p-value** | $\boldsymbol{\eta}_{\boldsymbol{p}}^{\boldsymbol{2}}$ |
| --- | --- | --- | --- | --- | --- | --- |
| **C3** | Delta | 2,3 ± 0,2 | 2,2 ± 0,3 | 3,68 | 0,07 | 0,11 |
|  | Theta | 1,9 ± 0,2 | 1,8 ± 0,3 | 2,50 | 0,12 | 0,08 |
|  | Alpha | 1,5 ± 0,2 | 1,4 ± 0,2 | 3,89 | 0,06 | 0,12 |
|  | **Beta** | 1,2 ± 0,2 | 1,1 ± 0,2 | 6,40 | 0,02* | 0,18^†^ |
|  | Total | 2,5 ± 0,2 | 2,4 ± 0,2 | 4,05 | 0,05 | 0,12 |
| **C4** | Delta | 2,3 ± 0,1 | 2,1 ± 0,2 | 3,10 | 0,09 | 0,09 |
|  | Theta | 1,9 ± 0,2 | 1,7 ± 0,3 | 2,79 | 0,11 | 0,09 |
|  | Alpha | 1,5 ± 0,2 | 1,4 ± 0,2 | 3,55 | 0,07 | 0,11 |
|  | **Beta** | 1,2 ± 0,2 | 1,1 ± 0,2 | 6,47 | 0,02* | 0,18^†^ |
|  | Total | 2,5 ± 0,1 | 2,4 ± 0,2 | 3,74 | 0,06 | 0,11 |
| F3 | Delta | 2,3 ± 0,2 | 2,2 ± 0,2 | 1,62 | 0,21 | 0,05 |
|  | Theta | 1,9 ± 0,2 | 1,8 ± 0,3 | 1,18 | 0,29 | 0,04 |
|  | Alpha | 1,5 ± 0,2 | 1,3 ± 0,2 | 2,22 | 0,15 | 0,07 |
|  | Beta | 1,3 ± 0,2 | 1,1 ± 0,3 | 2,33 | 0,14 | 0,07 |
|  | Total | 2,6 ± 0,1 | 2,5 ± 0,2 | 1,61 | 0,21 | 0,05 |
| F4 | Delta | 2,3 ± 0,2 | 2,2 ± 0,2 | 1,31 | 0,26 | 0,04 |
|  | Theta | 1,9 ± 0,2 | 1,8 ± 0,3 | 0,64 | 0,43 | 0,02 |
|  | Alpha | 1,4 ± 0,2 | 1,3 ± 0,2 | 1,88 | 0,18 | 0,06 |
|  | Beta | 1,3 ± 0,2 | 1,1 ± 0,3 | 1,53 | 0,23 | 0,05 |
|  | Total | 2,5 ± 0,1 | 2,5 ± 0,2 | 1,17 | 0,29 | 0,04 |
| F7 | Delta | 2,0 ± 0,1 | 2,0 ± 0,3 | 0,17 | 0,68 | 0,01 |
|  | Theta | 1,5 ± 0,1 | 1,4 ± 0,3 | 0,27 | 0,61 | 0,01 |
|  | Alpha | 1,1 ± 0,2 | 1,0 ± 0,2 | 1,20 | 0,28 | 0,04 |
|  | Beta | 0,9 ± 0,2 | 0,8 ± 0,3 | 1,02 | 0,32 | 0,03 |
|  | Total | 2,2 ± 0,1 | 2,2 ± 0,3 | 0,02 | 0,88 | 0,00 |
| F8 | Delta | 2,0 ± 0,1 | 2,0 ± 0,3 | 0,01 | 0,93 | 0,00 |
|  | Theta | 1,5 ± 0,2 | 1,4 ± 0,3 | 0,14 | 0,72 | 0,01 |
|  | Alpha | 1,1 ± 0,2 | 1,0 ± 0,2 | 0,48 | 0,50 | 0,02 |
|  | Beta | 0,9 ± 0,2 | 0,9 ± 0,2 | 0,44 | 0,51 | 0,01 |
|  | Total | 2,2 ± 0,1 | 2,2 ± 0,3 | 0,00 | 0,98 | 0,00 |
| FP1 | Delta | 2,1 ± 0,1 | 2,1 ± 0,2 | 0,11 | 0,74 | 0,00 |
|  | Theta | 1,6 ± 0,2 | 1,6 ± 0,3 | 0,04 | 0,85 | 0,00 |
|  | Alpha | 1,2 ± 0,2 | 1,2 ± 0,2 | 0,22 | 0,64 | 0,01 |
|  | Beta | 1,0 ± 0,2 | 0,9 ± 0,2 | 0,55 | 0,47 | 0,02 |
|  | Total | 2,3 ± 0,1 | 2,3 ± 0,2 | 0,04 | 0,85 | 0,00 |
| FP2 | Delta | 2,1 ± 0,1 | 2,1 ± 0,2 | 0,10 | 0,76 | 0,00 |
|  | Theta | 1,6 ± 0,2 | 1,6 ± 0,3 | 0,13 | 0,72 | 0,00 |
|  | Alpha | 1,2 ± 0,2 | 1,2 ± 0,2 | 0,30 | 0,59 | 0,01 |
|  | Beta | 1,0 ± 0,2 | 0,9 ± 0,2 | 0,62 | 0,44 | 0,02 |
|  | Total | 2,3 ± 0,1 | 2,3 ± 0,2 | 0,01 | 0,92 | 0,00 |
| **O1** | **Delta** | 2,4 ± 0,3 | 2,2 ± 0,3 | 5,20 | 0,03* | 0,16^†^ |
|  | **Theta** | 1,9 ± 0,2 | 1,7 ± 0,2 | 7,25 | 0,01* | 0,21^†^ |
|  | Alpha | 1,9 ± 0,2 | 1,7 ± 0,3 | 3,73 | 0,06 | 0,12 |
|  | **Beta** | 1,4 ± 0,1 | 1,2 ± 0,2 | 12,64 | 0,001* | 0,31^†^ |
|  | **Total** | 2,7 ± 0,2 | 2,5 ± 0,2 | 5,79 | 0,02* | 0,17^†^ |
| **O2** | **Delta** | 2,4 ± 0,3 | 2,2 ± 0,3 | 6,10 | 0,02* | 0,17^†^ |
|  | **Theta** | 1,9 ± 0,2 | 1,7 ± 0,2 | 7,25 | 0,01* | 0,21^†^ |
|  | Alpha | 1,9 ± 0,2 | 1,7 ± 0,3 | 3,73 | 0,06 | 0,12 |
|  | **Beta** | 1,4 ± 0,2 | 1,2 ± 0,2 | 9,60 | 0,004* | 0,24^†^ |
|  | **Total** | 2,7 ± 0,2 | 2,5 ± 0,2 | 6,16 | 0,02* | 0,17^†^ |
| P3 | Delta | 2,3 ± 0,2 | 2,2 ± 0,2 | 2,15 | 0,15 | 0,07 |
|  | Theta | 1,8 ± 0,2 | 1,8 ± 0,2 | 0,74 | 0,40 | 0,02 |
|  | Alpha | 1,7 ± 0,3 | 1,6 ± 0,3 | 0,56 | 0,46 | 0,02 |
|  | Beta | 1,3 ± 0,2 | 1,2 ± 0,2 | 2,14 | 0,15 | 0,07 |
|  | Total | 2,5 ± 0,2 | 2,4 ± 0,2 | 1,55 | 0,22 | 0,05 |
| **P4** | **Delta** | 2,3 ± 0,2 | 2,2 ± 0,2 | 5,24 | 0,03* | 0,15^†^ |
|  | Theta | 1,9 ± 0,2 | 1,7 ± 0,2 | 4,68 | 0,04 | 0,14 |
|  | Alpha | 1,7 ± 0,2 | 1,6 ± 0,3 | 1,94 | 0,17 | 0,06 |
|  | **Beta** | 1,3 ± 0,2 | 1,1 ± 0,2 | 7,79 | 0,01* | 0,21^†^ |
|  | **Total** | 2,6 ± 0,2 | 2,4 ± 0,2 | 5,50 | 0,03* | 0,16^†^ |
| T7 | Delta | 1,8 ± 0,1 | 1,8 ± 0,3 | 0,06 | 0,81 | 0,00 |
|  | Theta | 1,4 ± 0,1 | 1,3 ± 0,3 | 0,18 | 0,68 | 0,01 |
|  | Alpha | 1,2 ± 0,2 | 1,1 ± 0,3 | 1,55 | 0,22 | 0,05 |
|  | Beta | 0,9 ± 0,1 | 0,8 ± 0,3 | 2,11 | 0,16 | 0,07 |
|  | Total | 2,1 ± 0,1 | 2,1 ± 0,3 | 0,00 | 0,98 | 0,00 |
| T8 | Delta | 1,8 ± 0,2 | 1,8 ± 0,3 | 0,00 | 0,97 | 0,00 |
|  | Theta | 1,4 ± 0,2 | 1,3 ± 0,3 | 0,57 | 0,46 | 0,02 |
|  | Alpha | 1,1 ± 0,3 | 1,0 ± 0,3 | 1,10 | 0,30 | 0,04 |
|  | Beta | 0,9 ± 0,2 | 0,8 ± 0,2 | 2,31 | 0,14 | 0,08 |
|  | Total | 2,1 ± 0,1 | 2,0 ± 0,3 | 0,10 | 0,75 | 0,00 |
| P7 | Delta | 2,1 ± 0,3 | 2,0 ± 0,3 | 0,75 | 0,39 | 0,02 |
|  | Theta | 1,7 ± 0,2 | 1,6 ± 0,2 | 1,21 | 0,28 | 0,04 |
|  | Alpha | 1,5 ± 0,2 | 1,5 ± 0,3 | 0,56 | 0,46 | 0,02 |
|  | Beta | 1,2 ± 0,2 | 1,1 ± 0,2 | 2,38 | 0,13 | 0,07 |
|  | Total | 2,4 ± 0,2 | 2,3 ± 0,2 | 0,74 | 0,40 | 0,02 |
| **P8** | Delta | 2,1 ± 0,2 | 2,0 ± 0,3 | 1,70 | 0,20 | 0,05 |
|  | Theta | 1,7 ± 0,2 | 1,5 ± 0,2 | 2,96 | 0,10 | 0,09 |
|  | Alpha | 1,6 ± 0,2 | 1,4 ± 0,3 | 3,16 | 0,09 | 0,10 |
|  | **Beta** | 1,2 ± 0,2 | 1,0 ± 0,2 | 7,84 | 0,01* | 0,21^†^ |
|  | Total | 2,4 ± 0,2 | 2,3 ± 0,2 | 2,35 | 0,14 | 0,07 |

Results are presented as mean ± standard deviation. NT, Neurotypical; ASD, Autism spectrum disorder.* p <0.05; ^†^ $\eta_{p}^{2} \geq$0.14.

**Table S2.** Intrahemispheric proximal ratios differences between NT and ASD participants.

| **Electrode** | **Frequency band** | **NT** | **ASD** | **F-value** | **p-value** | $\boldsymbol{\eta}_{\boldsymbol{p}}^{\boldsymbol{2}}$ |
| --- | --- | --- | --- | --- | --- | --- |
| **F3-FP1** | **Delta** | 24,3 ± 7,5 | 11,4 ± 16,3 | 7,86 | 0,01* | 0,21^†^ |
|  | Theta | 35,3 ± 9,6 | 26,8 ± 18,5 | 2,49 | 0,13 | 0,08 |
|  | Alpha | 29,0 ± 7,0 | 20,2 ± 15,7 | 4,05 | 0,05 | 0,12 |
|  | Beta | 33,5 ± 8,2 | 23,4 ± 18,3 | 3,84 | 0,06 | 0,11 |
|  | **Total** | 28,1 ± 7,3 | 16,6 ± 17,0 | 5,94 | 0,02* | 0,17^†^ |
| **F4-FP2** | **Delta** | 20,4 ± 8,4 | 9,6 ± 14,2 | 6,65 | 0,02* | 0,18^†^ |
|  | Theta | 30,8 ± 10,5 | 26,7 ± 18,2 | 0,60 | 0,45 | 0,02 |
|  | Alpha | 26,1 ± 7,1 | 19,3 ± 14,0 | 2,83 | 0,10 | 0,09 |
|  | Beta | 30,1 ± 9,1 | 23,7 ± 16,0 | 1,89 | 0,18 | 0,06 |
|  | Total | 24,0 ± 8,2 | 15,4 ± 15,7 | 3,65 | 0,07 | 0,11 |
| F7-FP1 | Delta | -18,0 ± 5,1 | -15,4 ± 11,4 | 0,63 | 0,43 | 0,02 |
|  | Theta | -17,7 ± 5,9 | -18,4 ± 7,3 | 0,09 | 0,77 | 0,00 |
|  | Alpha | -12,4 ± 6,8 | -14,4 ± 8,0 | 0,55 | 0,46 | 0,02 |
|  | Beta | -10,2 ± 9,0 | -8,8 ± 10,0 | 0,16 | 0,70 | 0,01 |
|  | Total | -17,0 ± 4,5 | -15,2 ± 9,9 | 0,39 | 0,54 | 0,01 |
| F8-FP2 | Delta | -17,6 ± 7,0 | -18,9 ± 12,4 | 0,13 | 0,72 | 0,00 |
|  | Theta | -17,4 ± 8,2 | -17,7 ± 8,5 | 0,01 | 0,92 | 0,00 |
|  | Alpha | -11,9 ± 7,7 | -13,7 ± 7,8 | 0,44 | 0,51 | 0,02 |
|  | Beta | -8,0 ± 9,0 | -7,1 ± 6,5 | 0,10 | 0,76 | 0,00 |
|  | Total | -16,2 ± 7,1 | -17,0 ± 10,2 | 0,07 | 0,80 | 0,00 |
| C3-F3 | Delta | -2,7 ± 6,7 | -8,7 ± 10,4 | 3,63 | 0,07 | 0,11 |
|  | Theta | -5,0 ± 7,5 | -8,9 ± 9,8 | 1,62 | 0,21 | 0,05 |
|  | Alpha | 9,9 ± 5,1 | 5,0 ± 10,6 | 2,70 | 0,11 | 0,08 |
|  | Beta | -4,7 ± 9,5 | -6,7 ± 19,1 | 0,14 | 0,71 | 0,01 |
|  | Total | -1,8 ± 5,6 | -7,4 ± 10,1 | 3,60 | 0,07 | 0,11 |
| **F4-C4** | Delta | -3,3 ± 4,6 | -9,2 ± 10,8 | 3,86 | 0,06 | 0,11 |
|  | **Theta** | -4,2 ± 5,6 | -11,2 ± 11,2 | 4,77 | 0,04* | 0,14^†^ |
|  | Alpha | 9,5 ± 6,0 | 3,7 ± 10,9 | 3,37 | 0,08 | 0,10 |
|  | Beta | -4,2 ± 9,1 | -7,3 ± 21,1 | 0,28 | 0,60 | 0,01 |
|  | Total | -2,0 ± 4,1 | -8,5 ± 11,1 | 4,61 | 0,04* | 0,13 |
| T7-F3 | Delta | -54,5 ± 7,5 | -42,2 ± 23,3 | 3,58 | 0,07 | 0,11 |
|  | Theta | -55,7 ± 10,6 | -48,9 ± 23,2 | 1,02 | 0,32 | 0,03 |
|  | Alpha | -35,1 ± 13,7 | -30,8 ± 21,1 | 0,42 | 0,52 | 0,01 |
|  | Beta | -40,6 ± 19,4 | -32,7 ± 24,3 | 0,95 | 0,34 | 0,03 |
|  | Total | -52,1 ± 8,8 | -41,3 ± 22,7 | 2,80 | 0,11 | 0,09 |
| T8-F4 | Delta | -52,2 ± 6,3 | -43,3 ± 27,6 | 1,49 | 0,23 | 0,05 |
|  | Theta | -54,8 ± 11,1 | -53,6 ± 26,1 | 0,03 | 0,88 | 0,00 |
|  | Alpha | -36,3 ± 12,4 | -35,3 ± 21,5 | 0,03 | 0,87 | 0,00 |
|  | Beta | -41,5 ± 12,4 | -37,0 ± 24,5 | 0,40 | 0,53 | 0,01 |
|  | Total | -50,3 ± 7,4 | -44,0 ± 26,6 | 0,78 | 0,38 | 0,03 |
| **C3-F7** | **Delta** | 37,2 ± 10,5 | 18,1 ± 23,0 | 8,21 | 0,01* | 0,22^†^ |
|  | Theta | 45,1 ± 12,2 | 35,3 ± 24,5 | 1,84 | 0,19 | 0,06 |
|  | Alpha | 47,5 ± 7,6 | 37,4 ± 21,9 | 2,69 | 0,11 | 0,09 |
|  | **Beta** | 37,6 ± 8,5 | 25,1 ± 19,5 | 4,98 | 0,03* | 0,15^†^ |
|  | **Total** | 40,7 ± 9,8 | 24,1 ± 22,8 | 6,43 | 0,02* | 0,18^†^ |
| **C4-F8** | **Delta** | 33,8 ± 7,3 | 19,4 ± 25,3 | 4,53 | 0,04* | 0,13 |
|  | Theta | 42,5 ± 8,1 | 32,6 ± 25,6 | 2,07 | 0,16 | 0,06 |
|  | Alpha | 44,7 ± 7,8 | 34,7 ± 32,6 | 2,41 | 0,13 | 0,07 |
|  | Beta | 33,4 ± 10,8 | 22,4 ± 21,1 | 3,29 | 0,08 | 0,10 |
|  | **Total** | 37,1 ± 5,9 | 23,6 ± 24,7 | 4,24 | 0,05* | 0,12 |
| T7-F7 | Delta | -18,6 ± 7,9 | -19,5 ± 12,3 | 0,05 | 0,82 | 0,00 |
|  | Theta | -9,6 ± 8,2 | -9,1 ± 9,1 | 0,02 | 0,88 | 0,00 |
|  | Alpha | 4,5 ± 9,4 | 2,3 ± 10,4 | 0,38 | 0,54 | 0,01 |
|  | Beta | 0,2 ± 13,0 | -3,4 ± 16,3 | 0,45 | 0,51 | 0,02 |
|  | Total | -12,5 ± 6,8 | -13,4 ± 10,7 | 0,08 | 0,79 | 0,00 |
| T8-F8 | Delta | -19,2 ± 9,4 | -20,1 ± 17,8 | 0,03 | 0,86 | 0,00 |
|  | Theta | -12,5 ± 12,2 | -17,5 ± 11,5 | 1,40 | 0,25 | 0,04 |
|  | Alpha | 0,07 ± 12,0 | -4,5 ± 6,6 | 1,85 | 0,18 | 0,06 |
|  | Beta | -5,5 ± 10,5 | -10,8 ± 17,3 | 1,07 | 0,31 | 0,03 |
|  | Total | -14,4 ± 9,2 | -17,1 ± 14,4 | 0,39 | 0,54 | 0,01 |
| P3-C3 | Delta | -0,5 ± 16,1 | 1,2 ± 14,7 | 0,10 | 0,75 | 0,00 |
|  | Theta | -5,7 ± 17,6 | 0,6 ± 11,7 | 1,45 | 0,24 | 0,05 |
|  | Alpha | 14,0 ± 22,4 | 22,9 ± 11,0 | 2,11 | 0,16 | 0,07 |
|  | Beta | 1,6 ± 16,6 | 9,1 ± 16,4 | 1,66 | 0,21 | 0,05 |
|  | Total | 0,3 ± 17,2 | 5,0 ± 12,0 | 0,82 | 0,37 | 0,03 |
| P4-C4 | Delta | 5,9 ± 10,0 | 1,5 ± 12,5 | 1,16 | 0,29 | 0,04 |
|  | Theta | 1,6 ± 11,1 | 0,1 ± 11,0 | 0,15 | 0,70 | 0,01 |
|  | Alpha | 21,3 ± 10,5 | 23,9 ± 12,3 | 0,40 | 0,53 | 0,01 |
|  | Beta | 7,5 ± 11,4 | 6,9 ± 14,0 | 0,02 | 0,90 | 0,00 |
|  | Total | 7,2 ± 9,9 | 5,2 ± 11,5 | 0,28 | 0,60 | 0,01 |
| P7-C3 | Delta | -21,0 ± 18,7 | -14,5 ± 26,2 | 0,64 | 0,43 | 0,02 |
|  | Theta | -22,5 ± 19,4 | -18,4 ± 24,0 | 0,27 | 0,61 | 0,01 |
|  | Alpha | -0,3 ± 16,8 | 10,1 ± 16,4 | 3,13 | 0,09 | 0,10 |
|  | Beta | -7,9 ± 16,6 | -1,8± 19,9 | 0,87 | 0,36 | 0,03 |
|  | Total | -17,9 ± 17,8 | -10,2 ± 23,2 | 1,08 | 0,31 | 0,04 |
| P8-C4 | Delta | -16,8 ± 17,2 | -16,5 ± 28,5 | 0,002 | 1,00 | 0,00 |
|  | Theta | -19,6 ± 20,4 | -21,0 ± 27,3 | 0,03 | 0,87 | 0,00 |
|  | Alpha | 7,7 ± 16,8 | 6,5 ± 21,0 | 0,03 | 0,86 | 0,00 |
|  | Beta | -4,7 ± 18,1 | -8,0 ± 23,4 | 0,20 | 0,66 | 0,01 |
|  | Total | -13,3 ± 17,0 | -12,7 ± 26,6 | 0,01 | 0,95 | 0,00 |
| P3-T7 | Delta | 50,1 ± 20,3 | 36,9 ± 17,3 | 3,82 | 0,06 | 0,12 |
|  | Theta | 46,6 ± 23,3 | 43,1 ± 21,6 | 0,18 | 0,67 | 0,01 |
|  | Alpha | 52,2 ± 27,3 | 52,8 ± 21,1 | 0,004 | 0,95 | 0,00 |
|  | Beta | 37,4 ± 25,7 | 36,2 ± 16,4 | 0,03 | 0,87 | 0,00 |
|  | Total | 49,1 ± 22,3 | 40,2 ± 17,7 | 1,55 | 0,22 | 0,05 |
| **P4-T8** | **Delta** | 53,9 ± 7,7 | 37,9 ± 25,8 | 5,35 | 0,03* | 0,15^†^ |
|  | Theta | 52,9 ± 10,5 | 46,4 ± 25,5 | 0,84 | 0,37 | 0,03 |
|  | Alpha | 59,5 ± 11,7 | 55,0 ± 26,1 | 0,37 | 0,55 | 0,01 |
|  | Beta | 43,6 ± 15,1 | 38,6 ± 17,5 | 0,72 | 0,40 | 0,02 |
|  | Total | 53,8 ± 8,6 | 42,0 ± 24,8 | 3,07 | 0,09 | 0,09 |
| **P7-T7** | **Delta** | 34,5 ± 15,7 | 23,4 ± 13,4 | 4,52 | 0,04* | 0,14^†^ |
|  | Theta | 33,7 ± 12,6 | 26,3 ± 18,8 | 1,58 | 0,22 | 0,05 |
|  | Alpha | 44,0 ± 10,8 | 43,3 ± 19,1 | 0,01 | 0,91 | 0,00 |
|  | Beta | 30,9 ± 8,6 | 26,6 ± 14,4 | 0,94 | 0,34 | 0,03 |
|  | Total | 35,9 ± 13,3 | 27,2 ± 14,5 | 2,95 | 0,10 | 0,09 |
| **P8-T8** | **Delta** | 34,8 ± 14,2 | 23,1 ± 14,1 | 5,48 | 0,03* | 0,15^†^ |
|  | Theta | 34,6 ± 17,6 | 28,4 ± 20,3 | 0,85 | 0,36 | 0,03 |
|  | Alpha | 49,4 ± 15,0 | 42,6 ± 24,1 | 0,87 | 0,36 | 0,03 |
|  | Beta | 32,8 ± 18,4 | 25,6 ± 16,9 | 1,31 | 0,26 | 0,04 |
|  | Total | 36,8 ± 14,9 | 27,7 ± 16,1 | 2,69 | 0,11 | 0,08 |
| O1-P3 | Delta | 14,8 ± 23,6 | 5,2 ± 18,3 | 1,61 | 0,22 | 0,05 |
|  | Theta | 9,1 ± 24,1 | -2,8 ± 15,2 | 2,78 | 0,11 | 0,09 |
|  | Alpha | 22,5 ± 21,5 | 13,4 ± 12,9 | 2,10 | 0,16 | 0,07 |
|  | Beta | 12,1 ± 20,2 | 2,2 ± 12,9 | 2,66 | 0,11 | 0,09 |
|  | Total | 14,8 ± 22,7 | 5,1 ± 15,7 | 1,91 | 0,18 | 0,06 |
| O2-P4 | Delta | 13,8 ± 12,2 | 6,7 ± 17,5 | 1,72 | 0,20 | 0,05 |
|  | Theta | 5,9 ± 10,5 | 0,5 ± 14,7 | 1,37 | 0,25 | 0,04 |
|  | Alpha | 20,5 ± 13,6 | 14,4 ± 11,0 | 1,96 | 0,17 | 0,06 |
|  | Beta | 8,7 ± 12,5 | 4,9 ± 9,1 | 1,00 | 0,33 | 0,03 |
|  | Total | 13,0 ± 11,3 | 7,0 ± 14,8 | 1,66 | 0,21 | 0,05 |
| O1-P7 | Delta | 24,3 ± 25,7 | 20,8 ± 11,6 | 0,25 | 0,62 | 0,01 |
|  | Theta | 17,1 ± 22,8 | 16,3 ± 9,0 | 0,02 | 0,89 | 0,00 |
|  | Alpha | 24,1 ± 23,8 | 25,9 ± 13,7 | 0,07 | 0,80 | 0,00 |
|  | Beta | 11,9 ± 22,1 | 13,0 ± 8,7 | 0,04 | 0,85 | 0,00 |
|  | Total | 22,0 ± 24,3 | 20,1 ± 10,3 | 0,09 | 0,77 | 0,00 |
| **O2-P8** | **Delta** | 43,8 ± 21,3 | 25,4 ± 10,2 | 10,14 | 0,003* | 0,25^†^ |
|  | Theta | 29,6 ± 18,5 | 20,5 ± 11,4 | 2,89 | 0,10 | 0,09 |
|  | Alpha | 46,2 ± 31,7 | 36,5 ± 22,0 | 1,03 | 0,32 | 0,03 |
|  | Beta | 23,9 ± 21,3 | 19,6 ± 13,6 | 0,49 | 0,49 | 0,02 |
|  | **Total** | 39,7 ± 21,3 | 25,2 ± 12,0 | 5,81 | 0,02* | 0,16^†^ |

Results are presented as mean ± standard deviation. NT, Neurotypical; ASD, Autism spectrum disorder.* p <0.05; ^†^ $\eta_{p}^{2} \geq$0.14.

**Table S3.** Intrahemispheric distal ratios differences between NT and ASD participants.

| **Electrode** | **Frequency band** | **NT** | **ASD** | **F-value** | **p-value** | $\boldsymbol{\eta}_{\boldsymbol{p}}^{\boldsymbol{2}}$ |
| --- | --- | --- | --- | --- | --- | --- |
| O1-C3 | Delta | 13,9 ± 22,0 | 5,5 ± 23,6 | 0,99 | 0,33 | 0,03 |
|  | Theta | 3,5 ± 19,3 | -2,5 ± 22,2 | 0,62 | 0,44 | 0,02 |
|  | Alpha | 35,9 ± 15,7 | 34,8 ± 14,4 | 0,03 | 0,86 | 0,00 |
|  | Beta | 14,0 ± 14,5 | 11,1 ± 15,8 | 0,26 | 0,61 | 0,01 |
|  | Total | 15,0 ± 19,6 | 9,4 ± 20,9 | 0,55 | 0,46 | 0,02 |
| O2-C4 | Delta | 19,2 ± 18,1 | 7,5 ± 23,7 | 2,43 | 0,13 | 0,08 |
|  | Theta | 7,5 ± 17,7 | 0,4 ± 21,6 | 1,02 | 0,32 | 0,03 |
|  | Alpha | 39,3 ± 17,7 | 36,4 ± 16,5 | 0,23 | 0,64 | 0,01 |
|  | Beta | 15,7 ± 18,7 | 11,5 ± 14,7 | 0,51 | 0,48 | 0,02 |
|  | Total | 19,7 ± 17,5 | 11,5 ± 21,2 | 1,42 | 0,24 | 0,05 |
| **O1-T7** | **Delta** | 60,3 ± 16,0 | 41,3 ± 16,0 | 10,38 | 0,003* | 0,27^†^ |
|  | **Theta** | 55,8 ± 10,2 | 40,6 ± 19,2 | 6,70 | 0,02* | 0,19^†^ |
|  | Alpha | 68,9 ± 8,4 | 61,0 ± 20,0 | 1,78 | 0,19 | 0,06 |
|  | **Beta** | 49,0 ± 9,8 | 38,1 ± 14,9 | 5,19 | 0,03* | 0,16^†^ |
|  | **Total** | 60,5 ± 12,9 | 44,2 ± 15,4 | 9,45 | 0,01* | 0,25^†^ |
| **O2-T8** | **Delta** | 62,4 ±10,8 | 44,1 ± 16,9 | 12,98 | 0,001* | 0,30^†^ |
|  | Theta | 56,3 ± 14,4 | 46,7 ± 21,1 | 2,17 | 0,15 | 0,07 |
|  | Alpha | 70,2 ± 13,7 | 62,9 ± 23,7 | 1,09 | 0,30 | 0,04 |
|  | Beta | 49,3 ± 19,1 | 42,5 ± 17,8 | 1,09 | 0,30 | 0,04 |
|  | **Total** | 61,8 ± 11,7 | 47,7 ± 17,6 | 6,87 | 0,01* | 0,19^†^ |
| O1-F3 | Delta | 10,3 ± 23,1 | -2,2 ± 20,7 | 2,42 | 0,13 | 0,08 |
|  | Theta | -1,6 ± 17,6 | -11,1 ± 21,0 | 1,72 | 0,20 | 0,06 |
|  | Alpha | 43,5 ± 16,8 | 38,6 ± 16,5 | 0,64 | 0,43 | 0,02 |
|  | Beta | 9,2 ± 21,4 | 4,8 ± 28,0 | 0,22 | 0,64 | 0,01 |
|  | Total | 12,6 ± 20,3 | 2,7 ± 19,4 | 1,84 | 0,19 | 0,06 |
| **O2-F4** | **Delta** | 16,1 ± 18,7 | -0,8 ± 21,3 | 5,64 | 0,02* | 0,16^†^ |
|  | Theta | 3,4 ± 16,4 | -10,5 ± 21,2 | 4,26 | 0,05* | 0,12 |
|  | Alpha | 46,7 ± 18,7 | 39,0 ± 18,1 | 1,39 | 0,25 | 0,04 |
|  | Beta | 11,6 ± 23,1 | 4,3 ± 28,2 | 0,62 | 0,44 | 0,02 |
|  | **Total** | 17,9 ± 17,8 | 3,5 ± 19,2 | 4,76 | 0,04* | 0,14^†^ |
| **O1-F7** | **Delta** | 47,3 ± 18,0 | 24,1 ± 20,4 | 10,52 | 0,003* | 0,27^†^ |
|  | **Theta** | 48,6 ± 10,0 | 33,0 ± 18,0 | 7,89 | 0,01* | 0,22^†^ |
|  | Alpha | 70,8 ± 8,9 | 62,5 ± 18,4 | 2,28 | 0,14 | 0,08 |
|  | **Beta** | 49,1 ± 12,3 | 34,4 ± 20,4 | 5,21 | 0,03* | 0,16^†^ |
|  | **Total** | 51,9 ± 14,0 | 32,9 ± 18,4 | 9,57 | 0,004* | 0,26^†^ |
| **O2-F8** | **Delta** | 49,1 ± 13,6 | 26,9 ± 19,8 | 13,29 | 0,001* | 0,31^†^ |
|  | **Theta** | 47,8 ± 13,5 | 32,8 ± 18,1 | 6,93 | 0,01* | 0,19^†^ |
|  | Alpha | 70,0 ± 14,9 | 61,0 ± 21,9 | 1,80 | 0,19 | 0,06 |
|  | Beta | 45,4 ± 19,9 | 32,5 ± 21,3 | 3,12 | 0,09 | 0,09 |
|  | **Total** | 52,3 ± 13,0 | 34,2 ± 18,7 | 9,82 | 0,004* | 0,25^†^ |
| **O1-FP1** | **Delta** | 33,0 ± 18,5 | 9,5 ± 15,7 | 14,05 | 0,001* | 0,33^†^ |
|  | **Theta** | 33,3 ± 11,6 | 16,0 ± 18,7 | 8,56 | 0,01* | 0,23^†^ |
|  | Alpha | 63,9 ± 11,7 | 53,3 ± 20,0 | 2,88 | 0,10 | 0,09 |
|  | Beta | 40,2 ± 16,7 | 26,6 ± 21,8 | 3,49 | 0,07 | 0,11 |
|  | **Total** | 38,7 ± 14,7 | 19,2 ± 15,7 | 11,87 | 0,002* | 0,30^†^ |
| **O2-FP2** | **Delta** | 35,1 ± 15,0 | 8,8 ± 16,0 | 22,78 | 0,000* | 0,43^†^ |
|  | **Theta** | 33,7 ± 11,1 | 16,2 ± 19,6 | 9,35 | 0,01* | 0,24^†^ |
|  | Alpha | 64,5 ± 13,3 | 52,9 ± 20,8 | 3,40 | 0,08 | 0,10 |
|  | Beta | 39,9 ± 16,8 | 26,2 ± 22,6 | 3,67 | 0,07 | 0,11 |
|  | **Total** | 40,0 ± 12,6 | 18,8 ± 15,8 | 17,34 | 0,000* | 0,37^†^ |
| P7-F7 | Delta | 17,4 ± 18,3 | 4,4 ± 18,6 | 3,79 | 0,06 | 0,12 |
|  | Theta | 25,1 ± 13,0 | 17,8 ± 18,6 | 1,54 | 0,22 | 0,05 |
|  | Alpha | 47,2 ± 13,2 | 45,3 ± 17,8 | 0,10 | 0,75 | 0,00 |
|  | Beta | 30,5 ± 16,5 | 22,6 ± 22,3 | 1,20 | 0,28 | 0,04 |
|  | Total | 24,6 ± 15,1 | 14,5 ± 17,6 | 2,87 | 0,10 | 0,09 |
| **P8-F8** | **Delta** | 17,3 ± 16,2 | 3,2 ± 20,5 | 4,61 | 0,04* | 0,13 |
|  | Theta | 24,1 ± 15,8 | 11,8 ± 19,8 | 3,73 | 0,06 | 0,11 |
|  | Alpha | 49,7 ± 15,2 | 39,5 ± 22,4 | 2,22 | 0,15 | 0,07 |
|  | Beta | 28,4 ± 19,7 | 14,6 ± 23,8 | 3,13 | 0,09 | 0,09 |
|  | **Total** | 24,4 ± 14,8 | 11,2 ± 19,2 | 4,63 | 0,04* | 0,13 |
| P7-F3 | Delta | -23,5 ± 18,7 | -21,9 ± 22,9 | 0,04 | 0,84 | 0,00 |
|  | Theta | -27,0 ± 18,6 | -26,4 ± 22,7 | 0,01 | 0,93 | 0,00 |
|  | Alpha | 9,4 ± 18,1 | 14,8 ± 17,8 | 0,74 | 0,40 | 0,02 |
|  | Beta | -12,3 ± 22,2 | -7,5 ± 29,6 | 0,26 | 0,61 | 0,01 |
|  | Total | -19,6 ± 17,6 | -16,7 ± 21,3 | 0,17 | 0,69 | 0,01 |
| P8-F4 | Delta | -19,9 ± 17,0 | -24,3 ± 26,0 | 0,31 | 0,58 | 0,01 |
|  | Theta | -23,3 ± 20,2 | -30,8 ± 26,3 | 0,81 | 0,38 | 0,03 |
|  | Alpha | 16,9 ± 17,6 | 9,7 ± 21,3 | 1,05 | 0,31 | 0,03 |
|  | Beta | -8,5 ± 22,6 | -13,8 ± 31,2 | 0,29 | 0,59 | 0,01 |
|  | Total | -15,1 ± 17,0 | -20,3 ± 24,9 | 0,46 | 0,50 | 0,02 |
| P7-FP1 | Delta | 0,2 ± 17,1 | -11,1 ± 14,3 | 4,12 | 0,05 | 0,12 |
|  | Theta | 8,5 ± 14,6 | -0,1 ± 19,1 | 2,02 | 0,17 | 0,06 |
|  | Alpha | 36,9 ± 15,8 | 33,7 ± 17,9 | 0,29 | 0,59 | 0,01 |
|  | Beta | 21,2 ± 19,9 | 14,4 ± 24,3 | 0,73 | 0,40 | 0,02 |
|  | Total | 8,5 ± 14,9 | -0,4 ± 14,5 | 2,96 | 0,10 | 0,09 |
| **P8-FP2** | **Delta** | 0,1 ± 15,7 | -15,6 ± 19,0 | 6,44 | 0,02* | 0,18^†^ |
|  | Theta | 7,4 ± 15,4 | -5,7 ± 23,3 | 3,41 | 0,08 | 0,10 |
|  | Alpha | 40,9 ± 12,6 | 27,9 ± 21,8 | 4,06 | 0,05 | 0,12 |
|  | Beta | 21,5 ± 16,8 | 7,8 ± 25,8 | 3,07 | 0,09 | 0,09 |
|  | **Total** | 8,8 ± 13,7 | -5,6 ± 18,5 | 6,20 | 0,02* | 0,17^†^ |
| **P3-F7** | **Delta** | 35,5 ± 22,7 | 19,2 ± 14,8 | 5,79 | 0,02* | 0,17^†^ |
|  | Theta | 39,1 ± 26,1 | 35,9 ± 18,2 | 0,16 | 0,69 | 0,01 |
|  | Alpha | 55,2 ± 26,8 | 54,7 ± 18,9 | 0,003 | 0,96 | 0,00 |
|  | Beta | 33,6 ± 21,6 | 32,9 ± 21,5 | 0,386 | 0,54 | 0,01 |
|  | Total | 39,7 ± 23,6 | 28,7 ± 14,5 | 2,50 | 0,13 | 0,08 |
| **P4-F8** | **Delta** | 38,7 ± 9,1 | 20,5 ± 19,3 | 11,11 | 0,002* | 0,27^†^ |
|  | Theta | 43,8 ± 8,1 | 32,7 ± 20,6 | 3,84 | 0,06 | 0,11 |
|  | Alpha | 59,4 ± 12,6 | 52,5 ± 24,1 | 0,98 | 0,33 | 0,03 |
|  | Beta | 39,3 ± 15,8 | 28,5 ± 22,1 | 2,46 | 0,13 | 0,08 |
|  | **Total** | 43,0 ± 8,0 | 28,1 ± 20,3 | 7,05 | 0,01* | 0,19^†^ |
| P3-F3 | Delta | -3,1 ± 18,7 | -7,1 ± 14,4 | 0,46 | 0,50 | 0,02 |
|  | Theta | -10,4 ± 18,9 | -8,2 ± 12,9 | 0,15 | 0,71 | 0,01 |
|  | Alpha | 23,0 ± 24,5 | 27,3 ± 14,4 | 0,39 | 0,54 | 0,01 |
|  | Beta | -3,1 ± 19,6 | 3,3 ± 27,4 | 0,58 | 0,45 | 0,02 |
|  | Total | -1,4 ± 18,8 | -2,2 ± 12,6 | 0,02 | 0,89 | 0,00 |
| **P4-F4** | **Delta** | 2,6 ± 10,8 | -7,7 ± 8,6 | 8,90 | 0,01* | 0,23^†^ |
|  | Theta | -2,5 ± 11,9 | -11,1 ± 12,8 | 3,77 | 0,06 | 0,11 |
|  | Alpha | 29,9 ± 14,3 | 26,9 ± 15,8 | 0,30 | 0,59 | 0,01 |
|  | Beta | 3,3 ± 17,5 | 0,3 ± 25,9 | 0,15 | 0,71 | 0,01 |
|  | **Total** | 5,2 ± 10,6 | -3,3 ± 10,1 | 5,52 | 0,03* | 0,16^†^ |
| **P3-FP1** | **Delta** | 20,7 ± 22,0 | 4,2 ± 12,0 | 7,10 | 0,01* | 0,19^†^ |
|  | Theta | 25,1 ± 24,4 | 19,2 ± 16,9 | 0,64 | 0,43 | 0,02 |
|  | Alpha | 47,4 ± 25,2 | 44,2 ± 19,1 | 0,16 | 0,69 | 0,01 |
|  | Beta | 29,8 ± 19,8 | 25,1 ± 21,1 | 0,42 | 0,52 | 0,01 |
|  | Total | 26,1 ± 22,3 | 14,5 ± 12,2 | 3,47 | 0,07 | 0,10 |
| **P4-FP2** | **Delta** | 22,9 ± 9,7 | 1,9 ± 11,9 | 29,18 | 0,000* | 0,49^†^ |
|  | **Theta** | 28,6 ± 8,0 | 16,2 ± 18,4 | 5,84 | 0,02* | 0,16^†^ |
|  | Alpha | 51,8 ± 10,6 | 43,1 ± 21,7 | 1,98 | 0,17 | 0,06 |
|  | Beta | 33,0 ± 11,8 | 22,2 ± 22,4 | 2,77 | 0,11 | 0,09 |
|  | **Total** | 28,9 ± 7,4 | 12,1 ± 14,8 | 15,77 | 0,000* | 0,34^†^ |
| T7-FP1 | Delta | -35,3 ± 8,5 | -33,7 ± 13,7 | 0,14 | 0,71 | 0,01 |
|  | Theta | -26,6 ± 10,7 | -26,9 ± 12,4 | 0,003 | 0,96 | 0,00 |
|  | Alpha | -7,7 ± 13,9 | -11,9 ± 13,3 | 0,73 | 0,40 | 0,02 |
|  | Beta | -9,8 ± 18,9 | -12,0 ± 20,1 | 0,10 | 0,76 | 0,00 |
|  | Total | -28,7 ± 8,0 | -27,9 ± 12,6 | 0,05 | 0,83 | 0,00 |
| T8-FP2 | Delta | -35,4 ± 9,7 | -36,9 ± 19,7 | 0,07 | 0,80 | 0,00 |
|  | Theta | -29,0 ± 13,7 | -33,4 ± 17,1 | 0,66 | 0,42 | 0,02 |
|  | Alpha | -11,7 ± 11,9 | -17,9 ± 13,2 | 1,95 | 0,17 | 0,06 |
|  | Beta | -13,3 ± 12,5 | -17,4 ± 19,5 | 0,49 | 0,49 | 0,02 |
|  | Total | -29,8 ± 10,1 | -32,6 ± 17,6 | 0,31 | 0,58 | 0,01 |
| **C3-FP1** | **Delta** | 21,6 ± 10,8 | 3,4 ± 19,2 | 10,57 | 0,003* | 0,26^†^ |
|  | Theta | 30,7 ± 12,2 | 18,8 ± 22,7 | 3,27 | 0,08 | 0,10 |
|  | **Alpha** | 37,8 ± 6,7 | 24,8 ± 21,0 | 5,26 | 0,03* | 0,15^†^ |
|  | **Beta** | 29,3 ± 6,4 | 16,7 ± 20,2 | 5,32 | 0,03* | 0,15^†^ |
|  | **Total** | 26,3 ± 9,3 | 9,8 ± 19,7 | 8,81 | 0,01* | 0,23^†^ |
| **C4-FP2** | **Delta** | 17,2 ± 9,2 | 0,8 ± 18,3 | 9,86 | 0,004* | 0,25^†^ |
|  | Theta | 27,0 ± 11,4 | 16,3 ± 23,0 | 2,64 | 0,12 | 0,08 |
|  | **Alpha** | 34,7 ± 6,7 | 22,7 ± 20,2 | 4,84 | 0,04* | 0,14^†^ |
|  | Beta | 26,4 ± 7,6 | 15,8 ± 20,4 | 3,60 | 0,07 | 0,11 |
|  | **Total** | 22,1 ± 8,2 | 7,3 ± 19,1 | 7,72 | 0,01* | 0,21^†^ |

Results are presented as mean ± standard deviation. NT, Neurotypical; ASD, Autism spectrum disorder.* p <0.05; ^†^ $\eta_{p}^{2} \geq$0.14.

**Table S4.** Interhemispheric ratios differences between NT and ASD participants.

| **Electrode** | **Frequency band** | **NT** | **ASD** | **F-value** | **p-value** | $\boldsymbol{\eta}_{\boldsymbol{p}}^{\boldsymbol{2}}$ |
| --- | --- | --- | --- | --- | --- | --- |
| O1-O2 | Delta | -2,9 ± 7,4 | -0,5 ± 7,9 | 0,77 | 0,39 | 0,03 |
|  | Theta | -1,6 ± 7,4 | -0,5 ± 8,3 | 0,17 | 0,69 | 0,01 |
|  | Alpha | -4,4 ± 7,6 | 0,4 ± 9,3 | 1,55 | 0,22 | 0,05 |
|  | Beta | -1,6 ± 6,5 | -0,2 ± 8,0 | 0,24 | 0,63 | 0,01 |
|  | Total | -3,0 ± 6,6 | -0,4 ± 7,2 | 1,00 | 0,33 | 0,03 |
| P7-P8 | Delta | -1,3 ± 11,2 | 3,7 ± 12,7 | 1,39 | 0,25 | 0,04 |
|  | Theta | -0,7 ± 12,5 | 5,2 ± 15,7 | 1,33 | 0,26 | 0,04 |
|  | Alpha | -5,6 ± 16,9 | 4,9 ± 18,3 | 2,85 | 0,10 | 0,09 |
|  | Beta | -1,6 ± 16,6 | 6,0 ± 18,9 | 1,44 | 0,24 | 0,05 |
|  | Total | -2,0 ± 12,7 | 4,2 ± 13,7 | 1,74 | 0,20 | 0,06 |
| P3-P4 | Delta | -2,4 ± 18,8 | 1,2 ± 7,7 | 0,55 | 0,46 | 0,02 |
|  | Theta | -4,0 ± 20,4 | 2,9 ± 6,2 | 1,78 | 0,19 | 0,06 |
|  | Alpha | -4,3 ± 20,3 | 0,6 ± 11,4 | 0,73 | 0,40 | 0,02 |
|  | Beta | -3,5 ± 18,1 | 2,6 ± 9,5 | 1,45 | 0,24 | 0,05 |
|  | Total | -3,2 ± 19,2 | 1,5 ± 7,0 | 0,91 | 0,35 | 0,03 |
| T7-T8 | Delta | -0,6 ± 13,3 | 3,4 ± 20,1 | 0,40 | 0,53 | 0,01 |
|  | Theta | 1,5 ± 19,9 | 7,2 ± 22,4 | 0,56 | 0,46 | 0,02 |
|  | Alpha | 2,8 ± 24,2 | 5,4 ± 22,5 | 0,10 | 0,76 | 0,00 |
|  | Beta | 2,4 ± 27,5 | 5,0 ± 26,4 | 0,07 | 0,80 | 0,00 |
|  | Total | -0,1 ± 17,4 | 4,7 ± 20,7 | 0,48 | 0,50 | 0,02 |
| C3-C4 | Delta | 3,3 ± 4,4 | 1,3 ± 8,7 | 0,63 | 0,43 | 0,02 |
|  | Theta | 2,5 ± 3,6 | 2,4 ± 6,6 | 0,001 | 0,97 | 0,00 |
|  | Alpha | 2,4 ± 6,0 | 1,8 ± 9,4 | 0,05 | 0,83 | 0,00 |
|  | Beta | 2,0 ± 6,8 | 0,2 ± 9,5 | 0,36 | 0,55 | 0,01 |
|  | Total | 2,9 ± 3,9 | 1,6 ± 7,6 | 0,38 | 0,54 | 0,01 |
| F3-F4 | Delta | 2,7 ± 5,5 | 0,8 ± 6,6 | 0,77 | 0,39 | 0,03 |
|  | Theta | 3,2 ± 5,3 | 0,1 ± 6,2 | 2,35 | 0,14 | 0,07 |
|  | Alpha | 2,0 ± 5,7 | 0,5 ± 6,3 | 0,50 | 0,49 | 0,02 |
|  | Beta | 2,5 ± 6,8 | -0,8 ± 7,0 | 1,81 | 0,19 | 0,06 |
|  | Total | 2,8 ± 5,2 | 0,5 ± 6,0 | 1,31 | 0,26 | 0,04 |
| F7-F8 | Delta | -1,2 ± 9,0 | 2,5 ± 12,4 | 0,85 | 0,36 | 0,03 |
|  | Theta | -1,4 ± 11,7 | -1,0 ± 12,5 | 0,01 | 0,93 | 0,00 |
|  | Alpha | -0,9 ± 16,0 | -1,1 ± 16,0 | 0,001 | 0,97 | 0,00 |
|  | Beta | -2,8 ± 18,0 | -2,4 ± 15,8 | 0,01 | 0,94 | 0,00 |
|  | Total | -1,8 ± 10,2 | 1,0 ± 12,4 | 0,46 | 0,50 | 0,02 |
| FP1-FP2 | Delta | -1,3 ± 4,9 | -1,1 ± 3,6 | 0,02 | 0,90 | 0,00 |
|  | Theta | -1,8 ± 3,0 | -0,3 ± 4,2 | 1,34 | 0,26 | 0,04 |
|  | Alpha | -1,2 ± 4,3 | -0,4 ± 5,0 | 0,20 | 0,66 | 0,01 |
|  | Beta | -1,2 ± 4,3 | -0,7 ± 5,0 | 0,09 | 0,76 | 0,00 |
|  | Total | -1,6 ± 4,1 | -0,9 ± 3,7 | 0,28 | 0,60 | 0,01 |

Results are presented as mean ± standard deviation. NT, Neurotypical; ASD, Autism spectrum disorder.* p <0.05; ^†^ $\eta_{p}^{2} \geq$0.14.
